# Making the case: developing innovative adherence solutions for the treatment of tuberculosis

**DOI:** 10.1136/bmjgh-2018-001323

**Published:** 2019-02-01

**Authors:** Malvika Verma, Jennifer Furin, Robert Langer, Giovanni Traverso

**Affiliations:** 1 Department of Biological Engineering and Koch Institute for Integrative Cancer Research, Massachusetts Institute of Technology, Cambridge, Massachusetts, USA; 2 Department of Global Health and Social Medicine, Harvard Medical School, Boston, Massachusetts, USA; 3 Department of Chemical Engineering and Koch Institute for Integrative Cancer Research, Massachusetts Institute of Technology, Cambridge, Massachusetts, USA; 4 Division of Gastroenterology, Brigham and Women’s Hospital, Harvard Medical School, Boston, Massachusetts, USA; 5 Department of Mechanical Engineering, Massachusetts Institute of Technology, Cambridge, Massachusetts, USA

**Keywords:** tuberculosis, adherence, drug depot systems, gastric resident systems

Tuberculosis (TB), which claims the lives of over 3500 people every day, is the world’s leading killer among infectious diseases.^1^ According to the WHO, 10 million people developed TB in 2017 with a global economic burden amounting to $12 billion annually.^1 2^ Furthermore, TB is the most significant pathogen in the global antimicrobial resistance (AMR) crisis.^3^ Unless radical action is taken, drug-resistant strains of TB will account for 25% of the AMR-related deaths and cost the global economy $16.7 trillion by the year 2050.^3^ TB treatment is challenging with its prolonged and frequent dosing regimen that may be associated with challenging side effects.^4^ While significant work has been done to support adherence among people living with TB who are on treatment—including direct observation of therapy and provision of socioeconomic support—there has been limited focus on translation of how the medications themselves and their administration might be altered to improve adherence.

Technologies that enable extended drug release of medication have the potential to overcome patient non-adherence to long and frequent dosing regimens. Long-acting formulations are being implemented for the reduction in the frequency of HIV treatment administration, though they require injections which can be uncomfortable for patients.^5^ Instead, a long-acting oral dosage would be very attractive and improve adherence to treatment, as the oral route of drug delivery is preferred by patients. Novel ingestible gastric-resident systems for extended controlled drug release are being developed by several groups (including the Langer and Traverso laboratories) for antimalarials and antiretrovirals.^6 7^

The challenge with designing drug depot systems for TB treatment is to balance the ease and safety of administration with the accommodation of gram-level quantities of TB drugs which have low potency. Under the current regimen during the intensive phase, a 60 kg patient with TB swallows almost 100 g of antibiotics in 1 month.^8^ One potential area of development which could aid in improved delivery include inhaled or orally delivered nanocarriers which have been designed for extended release of existing TB drugs, although they have yet to be tested in large animal models.^9 10^ Considering that bedaquiline is the first new approved TB drug in more than 40 years and the dearth of others in the TB drug development pipeline to overcome challenges of the current drugs, nanotechnology can provide an enormous impact with design of novel and targeted delivery systems for existing drugs.^11^ Ideally, these nanomaterial-based systems would be inexpensive, easy to administer, minimise side effects and reduce the required dosing frequency to improve patient adherence.

Developments in depot systems and more potent drugs can also improve treatment of children, who comprised 1 million (10%) of the new TB cases in 2017.^1^ Children face challenges in adhering to their treatments due to the difficulty in swallowing pills, bad taste of crushed tablets and aversion to needles.^12^ Therefore, it is difficult for caregivers to ensure the child is achieving the correct dosage while minimising toxic effects. A recent study in Mozambique found that over 30% of children do not adhere to the WHO recommended regimen.^13^ Finally, child-friendly first-line TB formulations became available through the Global Drug Facility.^12^ Optimising second-line drugs for drug-resistant TB in children is much further behind, and there are currently few drug depot systems available to simplify treatment and improve adherence.^14 15^ Notably, a paediatric dispersible formulation of delaminid may be promising and is currently being assessed in clinical trials.^16^

TB treatment adherence challenges contribute to poor health outcomes, prolonged infectiousness, drug resistance, relapse and death. While most adherence work has focused on changing the behaviours of people taking TB medications, there has been little work done exploring how the medications might be altered to improve the experience of people living with TB. We challenge global health agencies and funding bodies to prioritise patient-friendly interventions that improve adherence by incentivising more collaborations between clinicians, engineers and patients. These include development of technologies to facilitate dose administration with more potent drugs or novel drug depot systems, while addressing the needs of vulnerable populations such as children. We recognise that preferences and adoption rates for drug delivery modalities, such as inhalable nanotechnology systems, transdermal patches, liquid formulations and gastric resident systems vary across patient groups (table 1). Increased interaction among physicians, engineers and the TB community stands to facilitate innovative solutions to maximise delivery of medicine to patients and transform the treatment of infectious diseases.

**Table 1:** Advantages and disadvantages of different routes of administration for drug delivery formulations relevant to infectious diseases

Route of administration	Site of absorption	Examples of drug delivery formulations	Advantages	Disadvantages
Enteral				
Oral (per os)	Along gastrointestinal tract	Ingestible gastric resident systems for antimalarials and antiretrovirals^6 7^	Non-invasive. Can be self-administered. Preferred by patients.	Non-adherence due to frequent dosing for high pill burden of TB treatment. Chemical environment is harsh. Degraded by first pass-metabolism.
		Solid lipid nanoparticles of TB treatment^9^		
		Paediatric dispersible tablets for Coartem and delaminid^16 17^		
Sublingual or buccal	Surfaces in the mouth	Metered sublingual spray of artemether (ArTiMist) for children^18^	Non-invasive. Can be self-administered. Rapid absorption. Avoids first-pass metabolism.	Low surface area for absorption which limits dose and may not be in line with gram-level dosing of TB treatment. Bitter taste of drugs. Prone to irritation of oral mucosa.
Rectal	Rectal mucosa	Rectal artesunate suppositories for the prereferral management of severe malaria^19^	Useful for unconscious patients and children. No need to taste-mask drug. Partial avoidance of first-pass metabolism.	Absorption can be slow or erratic. Frequent application to match gram-level dosing of TB treatment. Prone to irritation of rectal mucosa.
Parental				
Intravenous	Veins, systemic bioavailable	Artemisinin nanoformulation^20^	Achieves 100% bioavailability. Reproducible.	Invasive. Requires trained personnel. Prone to infection. Frequent injections to match gram-level dosing of TB treatment.
Intramuscular	Skeletal muscle	Nanoparticles of rilpivirine and cabotegravir for HIV treatment^21^	Rapid absorption. Avoids first-pass metabolism.	Invasive. Limited volume for injection, so may not match gram-level dosing of TB treatment. Risk of nerve damage.
		Atovaquone solid drug nanoparticles for malaria prophylaxis^22^		
Subcutaneous	Into tissue between dermis and muscle	Ultra-long-acting dolutegravir implant for HIV treatment and prevention^23^	Slow absorption and distribution compared with intramuscular. Avoids first-pass metabolism.	Invasive. Limited volume for injection, so may not match gram-level dosing of TB treatment. Risk of tissue damage.
		Nanochannel implant with refillable feature for delivery of tenofavir diphosphate^24^		
Intradermal	Into dermis layer	Intradermal injections of HIV DNA vaccines using needle-free injector^25^	Faster absorption and distribution compared with subcutaneous. Avoids first-pass metabolism. Higher immune responses for vaccinations.	Invasive. Limited volume for injection, so may not match gram-level dosing of TB treatment. Risk of tissue damage.
Intrathecal	Into cerebrospinal fluid	Intrathecal administration of isoniazid for TB meningitis treatment^26^	Bypasses blood–brain barrier. Local effect on meninges or cerebrospinal axis.	Invasive. Limited volume for injection, so may not match gram-level dosing of TB treatment. Risk of tissue damage.
Intra-articular	Into joint space	Intra-articular streptomycin in tuberculosis of the knee^27^	Avoids first-pass metabolism. Local effect on joint.	Invasive. Limited volume for injection, so may not match gram-level dosing of TB treatment. Risk of tissue damage.
Inhalation	Mucosal surfaces for the lung	Nebulised solid lipid nanoparticles for TB treatment^10^	Non-invasive. Large surface area for absorption. Avoids first-pass metabolism. Targets where TB bacteria reside.	Variability in dosing depends on patient technique. Requires portable, cheap and easy to operate devices for administration. Frequent inhalation to be compatible with gram-level dosing of TB treatment.
		Nano microparticle vaccine formulation for TB^28^		
Transdermal	Through skin	Film of HIV inhibitor IQP-0410^29^	Non-invasive. Can be self-administered. Avoids first-pass metabolism.	Transport barriers for many drugs. Slow absorption. May require frequent administration or very large patch to match gram-level dosing of TB.
		Solid dispersions of artemisinin for malaria treatment^30^		
Topical: ocular, nasal, skin	At site of application	Topical treatment of cutaneous TB using oil nanoemulsions^31^	Non-invasive. Can be self-administered. Rapid absorption. Local effect, so avoids side effects.	Transport barriers for many drugs. May require frequent administration to match gram-level dosing of TB.
Intravaginal	Mucosal surfaces lining the vagina	Monthly vaginal rings for dapivirine, an HIV drug^32^	Reduce frequency of dosing. Avoids first-pass metabolism. Dense network of blood vessels in vagina, so ideal for systemic drug absorption.	Invasive. Requires trained personnel. Implants may require frequent dosing to match gram-level dosing of TB treatment.
		Topical tenofovir disoproxil fumarate nanoparticles^33^		

TB, tuberculosis.
